# Monoclonal Antibodies Targeting Ion Channels and Their Therapeutic Potential

**DOI:** 10.3389/fphar.2019.00606

**Published:** 2019-06-05

**Authors:** Aurélien Haustrate, Aline Hantute-Ghesquier, Natalia Prevarskaya, V’yacheslav Lehen’kyi

**Affiliations:** ^1^Laboratory of Cell Physiology, INSERM U1003, Laboratory of Excellence Ion Channel Science and Therapeutics, Department of Biology, Faculty of Science and Technologies, University of Lille, Villeneuve d’Ascq, France; ^2^FONDATION ARC, Villejuif, France

**Keywords:** monoclonal antibody, ion channel, mechanism, targeting, therapy

## Abstract

Monoclonal antibodies (mAbs) represent a rapidly growing pharmaceutical class of protein drugs that becomes an important part of the precision therapy. mAbs are characterized by their high specificity and affinity for the target antigen, which is mostly present on the cell surface. Ion channels are a large family of transmembrane proteins that control ion transport across the cell membrane. They are involved in almost all biological processes in both health and disease and are widely considered as prospective targets. However, no antibody-based drug targeting ion channel has been developed so far that has progressed to clinical use. Thus, we provide a comprehensive review of the elaborated mAbs against ion channels, describe their mechanisms of action, and discuss their therapeutic potential.

## Monoclonal Antibodies and Ion Channels to Date

Since the first appearance of the therapeutic monoclonal antibody (mAb) (Orthoclone OKT3) in early 1986, this class of biopharmaceutical agents has significantly expanded, reaching more than 70 mAb products (76 for 2017, **Table 1**, inspired from: http://www.actip.org/products/monoclonal-antibodies-approved-by-the-ema-and-fda-for-therapeutic-use/), which were approved in the US or Europe for the treatment of various diseases and disorders (Ecker et al., 2015). If the approval rate will be the same, i.e., of approximately four to five new products per year, around 80 mAb drugs will appear on the market by 2020, and overall worldwide sales will be more than $125 billion (Shalini Shahani, 2017).

**Table 1:** Monoclonal antibodies (mAbs) approved by the EMA and FDA for therapeutic use.

International nonproprietary name (INN)	Target	Type	Year of first FDA approval	Therapeutic indication(s)
Avelumab	PD-L1	Human IgG1/κ	2017	Metastatic Merkel cell carcinoma
Dupilumab	IL-4Rα	Human IgG4	2017	Asthma; dermatitis
Durvalumab	PD-L1	Human IgG1/κ	2017	Metastatic urothelial carcinoma
Ocrelizumab	CD20	Humanized IgG1κ	2017	Multiple sclerosis
Brodalumab	IL-17RA	Human IgG2/κ	2017	Psoriasis
Bezlotoxumab	*C. difficile* toxin B	Human monoclonal antitoxin antibody	2016	Enterocolitis; pseudomembranous
Adalimumab	TNFα	Human IgG1	2016	Arthritis; juvenile rheumatoid arthritis; psoriatic arthritis; rheumatoid colitis; ulcerative Crohn’s disease; psoriasis; spondylitis; ankylosing
Reslizumab	IL-5	Human IgG4/κ	2016	Asthma
Olaratumab	PDGFR-α	Human IgG1	2016	Sarcoma
Obiltoxaximab	PA component of *B. anthracis* toxin	Chimeric (mouse/human) IgG1/κ	2016	Anthrax infection
Infliximab	TNFα	Chimeric human-murine IgG1	2016	Spondylitis; ankylosing; arthritis; rheumatoid colitis; ulcerative arthritis; psoriatic Crohn’s disease; psoriasis
Atezolizumab	PD-L1	Human IgG1	2016	Metastatic non-small cell lung cancer
Daratumumab	CD38	Human IgG1/κ	2015	Multiple myeloma
Elotuzumab	SLAMF7	Human IgG1	2015	Multiple myeloma
Necitumumab	EGFR	Human IgG1	2015	Carcinoma, non-small-cell lung
Secukinumab	Interleukin-17A	Human IgG1/κ	2015	Arthritis; psoriatic psoriasis; spondylitis; ankylosing
Mepolizumab	IL-5	Human IgG1/κ	2015	Asthma
Nivolumab	PD-1	Human IgG4	2015	Carcinoma; non-small-cell lung carcinoma; renal cell Hodgkin disease melanoma
Alirocumab	PCSK9	Human IgG1	2015	Dyslipidemias
Idarucizumab	Dabigatran etexilate	Human FaB	2015	Hemorrhage
Evolocumab	LDL-C/PCSK9	Human IgG2	2015	Dyslipidemias; hypercholesterolemia
Dinutuximab (1)	GD2	Human IgG1/κ	2015	Neuroblastoma
Bevacizumab	CD19	BiTEs	2014	Precursor cell lymphoblastic leukemia–lymphoma
Pembrolizumab	PD-1	Human IgG4	2014	Melanoma
Ramucirumab	VEGF	Human IgG1	2014	Stomach neoplasms
Vedolizumab	Integrin-α4β7	HumanizedIgG1	2014	Colitis; ulcerative Crohn’s disease
Siltuximab	cCLB8	Chimeric IgG1κ	2014	Giant lymph node hyperplasia
Alemtuzumab	CD52	Humanized IgG1	2014	Multiple sclerosis
Trastuzumab emtansine	HER2	Humanized IgG1 as ADC	2013	Breast cancer
Obinutuzumab	CD20	Humanized IgG1	2013	CLL
Raxibacumab	*Bacillus anthracis* protective antigen	Human IgG1	2012	Prevention and treatment of inhalation anthrax
Pertuzumab	HER2	Humanized IgG1	2012	Breast cancer
Infliximab	TNF-alpha	Chimeric IgG1 Ab	Not approved (approved in 1997 by EU EMA)	Spondylitis; ankylosing arthritis; rheumatoid colitis; ulcerative Crohn’s disease; arthritis; psoriatic psoriasis
Brentuximab	CD30 (conjugate of Mab and MMAE)	Chemeric IgG1 as ADC (antibody drug conjugate)	2011	Hodgkin lymphoma (HL), systemic anaplastic large cell lymphoma (ALCL)
Belimumab	BLyS	Human IgG1	2011	Systemic lupus erythematosus (SLE)
Ipilimumab	CTLA-4	Human IgG1	2011	Melanoma
Denosumab	RANKL	Human IgG2	2011	Prevention of SREs in patients with bone metastases from solid tumors
Tocilizumab	IL-6 receptor	Humanized IgG1	2010	Rheumatoid arthritis
Denosumab	RANKL	Human IgG2	2010	Osteoporosis
Ofatumumab	CD20	Human IgG1	2009	Chronic lymphocytic leukemia
Besilesomab	NCA-95	Murine IgG1	Not approved (approved in 1997 by EU EMA)	*In vivo* diagnosis of inflammation/infection sites *via* scintigraphic imaging → nontherapeutic
Canakinumab	IL-1β	Human IgG1	2009	Cryopyrin-associated periodic syndromes including familial cold autoinflammatory syndrome and Muckle–Wells syndrome; tumor necrosis factor receptor associated periodic syndrome (TRAPS); hyperimmunoglobulin D Syndrome (HIDS)/mevalonate kinase deficiency (MKD) and familial Mediterranean fever (FMF)
Golimumab	TNF*a*	Human IgG1	2009	Rheumatoid arthritis; psoriatic arthritis; ankylosing spondylitis
Ustekinumab	IL-12/IL-23	Human IgG1	2009	Plaque psoriasis
Certolizumab pegol	TNF*a*	Humanized IgG Fab fragment	2008	Crohn’s disease; rheumatoid arthritis
Catumaxomab	EpCAM and CD3	Trifunctional MAb IgG2a/IgG2b	Not approved	Malignant ascites in patients with EpCAM-positive carcinomas
Eculizumab	Complement C5	Humanized IgG2/4	2007	Paroxysmal nocturnal hemoglobinuria
Ranibizumab	VEGF-A	Humanized IgG1 Fab fragment	2006	Neovascular (wet) age-related macular degeneration; macular edema following retinal vein occlusion
Panitumumab	EGFR	Human IgG2	2006	Metastatic colorectal carcinoma
Catumaxomab	EpCAM	Humanized MAb	2005	Head and neck cancer
Natalizumab	VLA-4	Humanized IgG4	2004	Multiple sclerosis (relapsing); Crohn’s disease
Cetuximab	EGFR	Chimeric IgG1	2004	Head and neck cancer; colorectal cancer
Fanolesomab	CD15	Murine MAb	2004	Imaging of equivocal appendicitis → nontherapeutic
Bevacizumab	VEGF	Humanized IgG1	2004	Metastatic colorectal cancer; non-small cell lung cancer; metastatic breast cancer; glioblastoma multiforme; metastatic renal cell carcinoma
Omalizumab	IgE	Humanized IgG1	2003	Asthma
Tositumomab and iodine 131 tositumomab	CD20	Murine IgG2a	2003	Non-Hodgkin’s lymphoma
Efalizumab (2)	CD11a	Humanized IgG1	2003	Psoriasis
Ibritumomab tiuxetan	CD20	Murine IgG1	2002	Non-Hodgkin’s lymphoma
Adalimumab	TNFα	Human IgG1	2002	Rheumatoid arthritis; juvenile idiopathic arthritis; psoriatic arthritis; ankylosing spondylitis; Crohn’s disease, plaque psoriasis
Alemtuzumab	CD52	Humanized IgG1	2001	B-cell chronic lymphocytic leukemia
Gemtuzumab ozogamicin (3)	CD33	Humanized IgG4/toxin conjugate	2000	Acute myeloid leukemia (AML)
Trastuzumab	HER-2	Humanized IgG1	1998	Breast cancer; metastatic gastric or gastroesophageal junction adenocarcinoma
Infliximab	TNFα	Chimeric IgG1	1998	Crohn’s disease; ulcerative colitis; rheumatoid arthritis; ankylosing spondylitis; psoriatic arthritis; plaque psoriasis
Basiliximab	CD25 (a chain of IL2 receptor)	Chimeric IgG1	1998	Reversal of transplantation rejection
Palivizumab	F-protein of RS virus	Humanized IgG1	1998	Respiratory syncytial virus (RSV)
Necitumumab (4)	CD25 (a chain of IL2 receptor)	Humanized IgG1	1997	Reversal of transplantation rejection
Rituximab	CD20	Chimeric IgG1	1997	Non-Hodgkin’s lymphoma; chronic lymphocytic leukemia; rheumatoid arthritis
Votumumab (5)	Cytokeratin tumor-associated antigen		Not approved	Detection of carcinoma of the colon or rectum → nontherapeutic
Sulesomab	NCA90	Murine Fab fragment	Not approved	Diagnostic imaging forosteomyelitis → nontherapeutic
Arcitumomab (6)	Human CEA (carcinoembryonic antigen)	Murine Fab fragment	1996	Detection of colorectal cancer → nontherapeutic
Imiciromab (7)	Human cardiac myosin	Murine Fab fragment	1996	Myocardial infarction imaging agent → nontherapeutic
Capromab	Tumor surface antigen PSMA	Murine MAb	1996	Detection of prostate adenocarcinoma → nontherapeutic
Nofetumomab	Carcinoma-associated antigen	Murine Fab fragment	1996	Diagnostic imaging of small-cell lung cancer → nontherapeutic
Abciximab (8)	GPIIb/IIIa	Chimeric IgG1 Fab	1994	High-risk angioplasty (prevention of blood clots)
Satumomab	TAG-72	Murine MAb	1992	Detection of colorectal and ovarian cancers → nontherapeutic
Muromonab-CD3	CD3	Murine IgG2a	1986	Transplantation rejection

(1): Unituxin has been withdrawn from use in the European Union.

(2): Raptiva^®^ was approved in 2003 by the FDA and in 2004 by the EMA. It was voluntarily withdrawn from the market in EU in 2009 and in US in 2009.

(3): Mylotarg^®^ was approved in 2000 by the FDA. It was voluntarily withdrawn from the market in US in 2010.

(4): Zenapax^®^ was approved in 1997 by the FDA and in 1999 by the EMA. It was withdrawn from the market for commercial reasons in EU in 2009 and in US in 2009.

(5): Humaspect^®^ was approved in 1998 by the EMA. It was withdrawn from the market in EU in 2003.

(6): CEA-scan was approved in 1996 by the FDA and by the EMA. It was withdrawn from the market in EU in 2005.

(7): Myoscint^®^ has been discontinued.

(8): Country-specific approval (prior to EMA Centralized Procedure).

PD-L1, Programmed death-ligand 1; IgG1, immunoglubulin G, subclass 1; IL-4Rα, interleukin 4 receptor alpha; IgG4, immunoglubulin G, subclass 4; CD20, B-lymphocyte antigen CD20; IL-17RA, interleukin 17 receptor A; TNFα, tumor necrosis factor alpha; IL-5, interleukin 5; PDGFR-α, platelet-derived growth factor receptor A; PA, protective antigen; CD38, cluster of differentiation 38; SLAMF7, SLAM family member 7; EGFR, epidermal growth factor receptor; PD-1, programmed cell death protein 1; PCSK9, proprotein convertase subtilisin/kexin type 9; FaB, fragment antigen-binding; LDL-C, low-density lipoprotein; PCSK9, proprotein convertase subtilisin/kexin type 9; GD2, disialoganglioside; BiTEs, bi-specific T-cell engagers; VEGF, vascular endothelial growth factor; CD2, cluster of differentiation 2; CD3, cluster of differentiation 3; IgG2a, immunoglubulin G, subclass 2, isotype a; TAG-72, tumor-associated glycoprotein 72; MAb, monoclonal antibody; GPIIb/IIIa, glycoprotein IIb/IIIa; IgG1, immunoglubulin G, subclass 1; PSMA, prostate-specific membrane antigen; NCA90, membrane glycoprotein p95; CD25, interleukin-2 receptor alpha chain; RS, retinoschisin; IL2, interleukin 2; HER-2, human epidermal growth factor receptor 2; CD33, sialic acid binding Ig-like lectin 3; CD52, cluster of differentiation 52; EGFR, epidermal growth factor receptor; VlLA-4, Very Late Antigen-4; EpCAM, epithelial cell adhesion molecule; IL-12, interleukine 12; IL-23, interleukine 23; IL-1β, interleukine 16; RANKL, receptor activator of nuclear factor kappa-B ligand; CTLA4, cytotoxic T-lymphocyte-associated protein 4; BLyS, B lymphocyte stimulator; MMAE, monomethyl auristatin E; LDL-C, low-density lipoprotein; PCSK9, proprotein convertase subtilisin/kexin type 9; SLAMF7, SLAM family member 7; SLAMF7, SLAM family member 7; SRE, skeletal-related events; CLL, chronic lymphocytic leukemia; EMA, European Medicines Agency; FDA, Food and Drug Administration.

Ion channels are considered as attractive targets since they are present on the plasma membrane and are potentially accessible by the soluble membrane-impermeable therapeutic drugs such as antibodies. They also constitute the third bestselling group of prescribed drugs (Wickenden et al., 2012). Irrespective of this apparent success in the use of pharmacological tools against ion channels, only some of the predicted in the human genome 400 ion channel genes have been targeted to date (Bagal et al., 2013). Despite the advantage of the targeting ion channels using antibodies because of their high specificity, unfortunately, no antibody-based drug has been developed to date and used in clinics, though some of them have already been engaged in clinical trials and are discussed below.

## Ion Channels as Prospective Therapeutic Targets

There is no biological process to mention that does not involve an ion channel or ion flux. Ion channels constitute a wide family of transmembrane proteins that allow ion flux inside and outside of the cell. They differ by their structure, their specificity for ion, and also their activation mode and mode of action. Indeed, some of them are highly specific for a particular ion, such as calcium for transient receptor potential vanilloid family member 6 (TRPV6), or sodium for Nav1.7, while others allow the flux of all the ions or just cations. Moreover, they can be activated by many stimuli like ligand binding, voltage change, osmotic change, temperature, and so on (Clapham et al., 2001). According to the activation mode and where they are expressed, we can speculate their function in the body. Actually, ion channels participate in lots of cellular process: contraction, secretion, apoptosis, migration, gene transcription, and so forth. Thus, it is not surprising to find them involved in numerous physiopathologies like cancer, pain, autoimmune diseases, and cystic fibrosis (Smani et al., 2015). Moreover, these proteins constitute a preferential target for the development of therapeutic agents since they are present on the surface of the plasma membrane and are potentially accessible by the soluble membrane-impermeable therapeutic drugs such as antibodies.

Several treatments targeting ion channels have been developed but still show a lack of specificity and side effects (Lynch et al., 2017). A novel therapeutic approach to answer this issue is mAb. However, nowadays, only few mAbs have been generated (Wilkinson et al., 2015). In this article, we review the already published mAbs targeting the extracellular region of ion channels classified according to their ion selectivity and highlight their potential application in human therapeutics.

## Calcium Channels

The voltage-gated calcium channels constitute a wide range of channels that are mostly permeable for calcium and largely expressed in excitable cells such as neurons and muscles. They are activated by a depolarization of the plasma membrane and are involved in a lot of cellular functions like muscular contraction, neuronal excitation, modulation of gene expression, release of hormones or neurotransmitters, and so on. This family is composed of several types of voltage-gated calcium channels (L, P, N, R, and T type) differentiated by voltage, their preferential localization, and subunits composition (Catterall and Swanson, 2015). They are all similar, but display structural and pharmacological differences.

In 2013, a 1B50-1 mAb was reported, which specifically binds to the α2δ1 subunit (isoform 5) of voltage-gated calcium channels that is crucial in maintenance of tumor-initiating cell (TIC) properties in the case of hepatocellular carcinoma, which is a common malignancy worldwide, having poor prognosis and limited therapeutic efficacy (Lee et al., 2013). It has been shown that this mAb detects TICs of hepatocellular carcinoma with stem-cell-like properties (Zhao et al., 2013). These α2δ1 subunits, *via* modulating calcium oscillation amplitude, allow the impact of L-type and N-type calcium channel-mediated Ca^2+^-signalization [such as phosphorylation of extracellular signal-regulated kinases 1/2 (ERK1/2)] and thereby maintain the properties of TICs. Interestingly, the use of this mAb, in addition to be a functional liver TIC marker, has therapeutic properties on these cells by eliminating TICs and suppressing sphere (10 μg/ml) formation *in vitro* (a marker of cancer stem cell and aggressivity). In fact, by using terminal deoxynucleotidyl transferase (dUTP) nick-end labeling (TUNEL) assay, they have demonstrated that 1B50-1 treatment induced apoptosis of 1B50-1^+^ cells. Moreover, 1B50-1 treatment by intraperitoneal injection reduced tumor growth and increased TICs death *in vivo*. The data demonstrated that 1B50-1 has a therapeutic effect on hepatocellular carcinoma by targeting TICs, though it is always difficult to obtain complete pharmacokinetic control *in vivo* with an antibody alone, most likely because of inefficient penetration of the antibody to the cells inside the tumor mass and because of the presence of other transit-amplifying tumorigenic cells (Zhao et al., 2013). Thus, this antibody constitutes a promising therapy to target cells involved in the recurrence of hepatocellular carcinoma.

**Figure 1:**
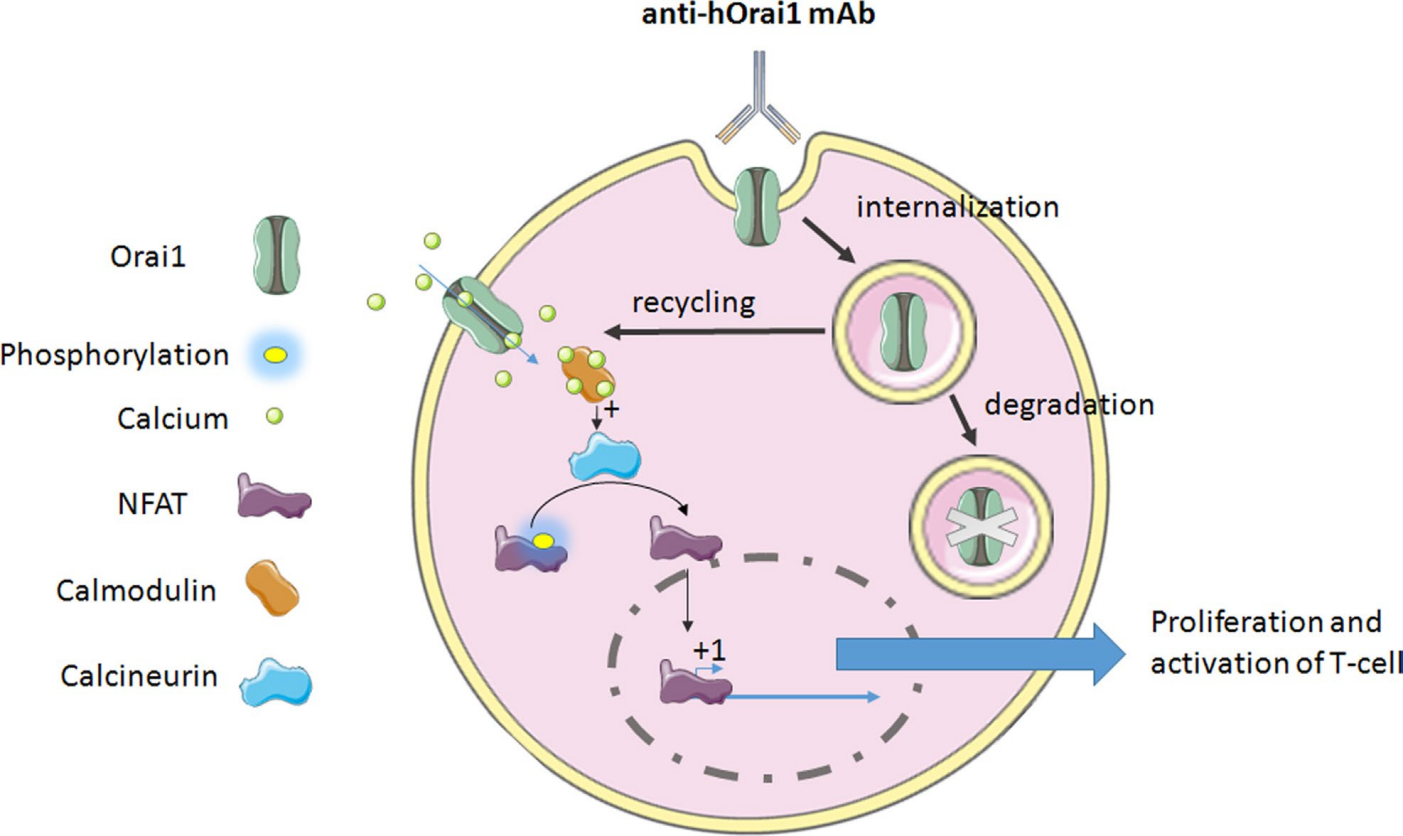
The mechanism of anti-hOrai1 channel monoclonal antibody (mAb) action on the human T-cell. mAb binds to the Orai1 extracellular domain and induces either internalization, which may lead to the channel recycling, or it completes degradation *via* the autophagy pathway. On the other hand, the inhibition of Ora1 channel was demonstrated by decrease in calcium flux. This decrease in calcium flux will affect Ca^2+^-calmodulin binding, leading to the inhibition of calcium-dependent phosphatase calcineurin. Dephosphorylation of NFAT (nuclear factor of activated T-cells) is a required event for its translocation to the nucleus and activation of gene transcription regulated by NFAT. In both cases, i.e., inhibition or internalization of Orai1, this binding of antibody provokes the impairment of Orai1-mediated signaling, leading to the inhibition of T-cell proliferation and activation, and cytokine production as well.

T-cell activation, proliferation, and cytokine production require Orai1-mediated calcium signaling (Feske et al., 2006; Gwack et al., 2008). Cox and collaborators have developed a specific anti-human Orai1 mAb, Orai1, a reactive clone, 10F8, targeting the second extracellular loop of the protein expressed on the lymphocytes (Cox et al., 2013). This mAb leads to the reduction of calcium flux through the internalization of the channel in lymphocytes (**Figure 1**). Actually, it is the only mechanism of internalization or retrograde trafficking shown so far. This process usually includes a dynein motor, which is strongly implicated in the retrograde trafficking of ion channels in endosomes from the cell surface (Choi et al., 2005), and leads to either degradation or recycling (Balse and Boycott, 2017).

Thus, anti-Orai1 mAb leads to an inhibition in T-cell proliferation and cytokine production *in vitro*. Orai1 was shown to be overexpressed in rheumatoid arthritis, a long-term autoimmune disease causing inflammation, swelling, and pain in and around the joints and other body organs (Liu et al., 2014), where the suppression of T-cell proliferation and cytokine production could be highly beneficial. This mAb has been tested on the cells isolated from rheumatoid arthritis patients and has shown the same efficacy. The same results were observed following the treatment [intraperitoneal (i.p) injection 10 mg/kg three times per week] *in vivo*, in a human T-cell-mediated graft-versus-host disease (GvHD) mouse model (Cox et al., 2013), suggesting its potential use for the human therapy *in vivo*.

Another team has generated high-affinity 100% human mAbs to human Orai1. This mAb binds to hOrai1 in its native conformation by targeting the extracellular loop 2 [notably, amino acids 210 to 217 of the hOrai1 extracellular loop 2 (ECL2) region] and displays an inhibitory activity (Lin et al., 2013). This leads to the inhibition in a dose-dependent manner of the current mediated by Orai1 and so that of the store-operated Ca^2+^ influx, seen in the whole-cell configuration patch clamp. Hence, the signal that comes out from this current is impaired. Indeed, NFAT transcription, studied by NFAT-Luciferase Reporter Assay, and cytokine release (IL-2 and IFN-*γ*) are disturbed too (**Figure 1**). In autoimmune disease, the use of such an mAb could be a novel therapeutic approach to reduce T-cell response and delay the manifestation of the disease.

Anti-Orai1 mAb from Cox and collaborators was used at 2–3 µg/ml for *in vitro* tests and binding to primary immune cells [cluster differentiation (CD)3+CD4+, CD3+CD8+], and CD19+) from the peripheral blood of a healthy individual using 12.5 µg/mL antibody. Meanwhile, the *in vivo* dose of the treated humanized NOD/Shi-scid/IL-2Rγnull (NOG) mice was 10 mg/kg, while the antibody from Lin and collaborators was used at ∼500 nM (75 µg/ml) and ∼0.05 µg/ml for the study of inhibitory concentration 50 (IC50) of anti-Orai1 mAbs in inhibiting interleukin 2 (IL-2) and interferon gamma (IFNγ) release in human whole blood (corresponding to ∼32 µg/kg of the average 60-kg human). These data can be compared to those from the use of anti-tumor necrosis factor alpha (TNFα) mAb, infliximab, which are of 10 or 50 mg/kg in preclinical assays in rodents (Derzi et al., 2016), and later in humans from 3 to 5 mg/kg for rheumatoid and psoriatic arthritis as intravenous (i.v.) injections (www.medscape.com).

## Nonselective Cation Channels

Transient receptor potential ankyrin 1 (TRPA1) is a nonselective cation channel that is expressed in nerve terminals innervating the skin, and more precisely in neurons of the dorsal root and trigeminal ganglia (Elitt et al., 2006; Obata et al., 2005). This channel is involved in pain. Indeed agonists such as mustard oil drive pain or neurogenic inflammation, both in human and in rodents (Bandell et al., 2004; Jordt et al., 2004). Additionally, a gain-of-function mutation in TRPA1 was linked to familial episodic pain syndrome by a human genetics study (Kremeyer et al., 2010). Familial episodic pain syndrome is a rare, genetic, peripheral neuropathy disorder characterized by recurrent, stereotyped, episodic intense pain, occurring predominantly in either the upper body or lower limbs in several members of a family, which is triggered or exacerbated by fatigue, cold exposure, fasting, weather changes, and/or physical stress (www.orpha.net). This channel was also strongly suspected to play a role in pathophysiologies like asthma, itch, and cough (Geppetti et al., 2010; Taylor-Clark et al., 2009; Viana and Ferrer-Montiel, 2009).

TRPA1 antagonist could be considered as potential therapeutics in these pathologies. In this context, mAbs to human TRPA1 have been generated. Of them, mAb 2B10 is the most potent. Like the others, it acts as an antagonist in a concentration-dependent manner of multiple modes of TRPA1 activation [allyl isothiocyanate (AITC), cold, and osmotic change]. For AITC and cold (4°C) TRPA1 activation, IC_50_ values of approximately 260 and 90 nM for either flow cytometry assay or agonist-induced ^45^Ca^2+^ uptake assays, respectively, are obtained. It also blocked TRPA1 activation by osmotic change *via* hypotonicity with IC_50_ values of 350 ± 60 nM, as well as activation by an endogenous agonist (4-oxo-2-nonenal). The maximal inhibition observed in the AITC-induced ^45^Ca^2+^ uptake assay of the TRPA1-mediated current was approximately 70%. The well-known antagonist, AMG9090 (acting *via* covalent modification of cysteine residues), totally blocked it, and thus is more effective than this mAb. The authors state that mAbs were not able to completely abolish AITC-induced TRPA1 activation (70% only), suggesting that 2B10 and 2D1 may only partially stabilize the channel conformation in the closed state, although the possibility that the mAbs act as partial pore blockers of TRPA1 cannot be completely excluded. This fact compromises the possibility to develop it as a therapeutic perspective (Lee et al., 2014b).

The nonselective cation channel TRPV1 (transient receptor potential vanilloid type 1) has several activation modes. Indeed, it can be activated by chemical ligands (capsaicin, anandamide, and acidity) and heat (>42°C); thus, it plays a role of integrator of multiple noxious stimuli and so is important in nociception (Caterina et al., 1999; Davis et al., 2000). Its role in clinical pain is clearly established, while the use of TRPV1 antagonist inhibits bone cancer pain (Ghilardi et al., 2005), which is common in patients with advanced breast, prostate, and lung cancer (Jimenez-Andrade et al., 2010), as well as in hyperalgesia in models of inflammatory pain (Honore et al., 2005).

In the other study by Klionsky and collaborators, an mAb binding to a small epitope of 39 amino acids, which corresponds to the entire prepore region of human TRPV1 (Thr^598^–Cys^636^), has been generated (Klionsky et al., 2006). A rabbit anti-rat TRPV1 polyclonal antibody (Ab-156H) acted as a full antagonist of proton activation (IC_50_ values for pH 5 and 5.5 were 364.68 ± 29.78 and 28.31 ± 6.30 nM, respectively) and as a partial antagonist of capsaicin, heat, and pH 6 potentiated chemical ligand (anandamide and capsaicin) activation (50–79% inhibition). Binding of the mAb to TRPV1 did not block the activation by either capsaicin or protons and, consistently with it, had no consequences on the stability of the channel conformation as shown by agonist-induced ^45^Ca^2+^ uptake assays on Chinese hamster ovary (CHO) cells expressing TRPV1. On the contrary, rabbit polyclonal antibodies against rat and human TRPV1 prepore region seem to partially lock or stabilize the channel in the closed state (Klionsky et al., 2006).

## Connexins

Connexins are proteins consisting of the assembly of six units, which form hemichannels at the cell surface. This structure can itself be active and allow exchanges between cytoplasm and extracellular space such as ion flux or allow the transfer of small molecules lower than 1–2 kDa, or usually form a gap junction by dimerization (Oshima, 2014; Revel et al., 1985). Both play a role in several physiological and pathological responses. The specific targeting of one or the other would allow one to distinguish the specific role of each of them. Indeed, despite the ubiquitous expression of connexins, some subtypes, such as 21 in humans, display changes in their expression in some tissues or tumors (Saez et al., 2003).

A specific Cx43 antibody [Connexin 43(E2) antibody], has been developed by Baklaushev and collaborators, which targets the second extracellular loop of Cx43 (Baklaushev et al., 2009). Initially, it was used for immunofluorescent analysis using preparations of both fixed and living cultures of rat, human glioma cells, and rat brain with experimental glioma. It was shown to specifically detect connexin hemichannels on the cell membranes and not dimer connexins, characteristic of gap junctions.

Connexin 43(E2) antibody prevents the opening of Cx43 hemichannels and thus blocks the hemichannels. Moreover, it prevents docking interaction, inhibits N-cadherin interaction, and reduces gap junction plaque size (Bao et al., 2011). The authors found that treatment with anti-Cx43 E2 (112 μg/ml), which suppresses Cx43 docking, significantly inhibited the kinetics of human KGN granulosa cells and normal human fibroblast self-assembly compared to the preimmune sera control (41.1 ± 4.5% and 24.5 ± 10.4% at 8 h, respectively).

Additionally, while blocking Cx43 hemichannels, it disturbed cell–cell communication, and could, by this way, prevent the release of small molecules such as prostaglandins and thus impair cell function (Siller-Jackson et al., 2008). Moreover, this mAb could be labeled with the fluorescent molecules and used for detection and staining of tumor cells highly expressing Cx43 on the cell surface, such as glioma cells. Recent findings demonstrate that connexins play an important role in the microenvironment of malignant glioma and that Cx43 is capable of conferring chemotherapeutic resistance to glioblastoma cells (Grek et al., 2018).

## P2XR Receptor Cation Channel Family

The P2X receptor family, also named adenosine 5′-triphosphate (ATP)-gated P2X receptor cation channel family, is composed of monovalent cation-permeable ligand-gated ion channels to be activated by extracellular adenosine 5′-triphosphate (ATP) (Valera et al., 1994). This family is encoded by seven genes in vertebrates, and they are largely distributed in the body. Some of them are implicated in pain feeling owing to the fact that they are predominantly expressed in nociceptive sensory neurons (Chen et al., 1995). Indeed, under pathological conditions, an increase in extracellular ATP leads to P2X receptor (P2XR) activation and pain signaling initiation (North, 2002). Thus, they constitute selective targets in some disorders such as neuropathic pain, which is pain caused by the damage or disease affecting the somatosensory nervous system and are usually associated with the abnormal sensations called dysesthesia or pain from normally nonpainful stimuli, i.e., allodynia.

mAbs against several isoforms of P2X receptor were developed. In their study, Shcherbatko and collaborators generated several mAbs directed against human P2X3. Depending on the homomeric or heteromeric composition of receptor (homomeric P2X3 or heteromeric P2X2/P2X3 receptors), as well as on its kinetic state and the duration of mAb action, different functional effects were observed (Shcherbatko et al., 2016). For example, 12D4 antibody had an IC_50_ of 16 nM while acting on an hP2X3 receptor for up to 18 min, while monoclonal hP2X3 antibodies inhibited recombinant rat P2X3 receptors, giving an IC_50_ of 185.5 ± 41.7. In such a way, mAb binds to the hP2X3 channel in its inactivated state and inhibits its activity. Surprisingly, with the same short-term exposure, this antibody potentiated the slow inactivated current mediated by the heteromeric human P2X2/P2X3 channel. Positive effects of mAb12D4 in a rat model of inflammatory pain suggest that mAb against P2X3 would be a prospective therapy in visceral pain symptoms, where pain occurs when pain receptors in the pelvis, abdomen, chest, or intestines are activated and may be accompanied by symptoms such as nausea, vomiting, changes in vital signs, as well as emotional manifestations (Sikandar and Dickenson, 2012).

An mAb against P2X4 receptor was also generated (Igawa et al., 2013). P2X4 receptor is known to be involved in neuropathic pain and notably in the allodynia after peripheral nerve injury (Nasu-Tada et al., 2006). This antibody has a strict recognition for P2X4 receptor including S–S formation in the extracellular domain. This was proved while detecting and immunoprecipitating rat P2X4 receptors in cultured cells. Such an mAb can be used as a tool in order to detect an increasing expression of P2X4 receptors related to increasing intensity of neuropathic pain (Igawa et al., 2013).

Finally, a 150-kDa specific mAb for human P2X7 receptor was generated (Buell et al., 1998). Its specificity of binding has been assessed in immunohistochemical studies on human lymphoid tissue by both immunoprecipitation and also by flow cytometry using XS63-transfected cells and human-blood-derived macrophage cells such as monocytes, known to express natively P2X7 receptors. The analysis of binding to HEK293 cells expressing other P2Xs such as P2X1 allows one to demonstrate the high specificity of the binding for the P2X7 receptor. This antibody acts as a selective antagonist of this receptor as shown by reduced response to a brief application of 2′,3′-(4-benzoyl)-benzoyl-ATP in cells expressing human P2X7 demonstrated in whole-cell configuration of the patch clamp. Indeed, a significantly reduced release of interleukin-1β was observed in THP-1 cells. In addition, this mAb successfully detects endogenous receptor with a high specificity (the estimated IC_50_ value was about 5 nmol/L) and can be used for the detection in Western blotting. The data also suggested that the antibody recognizes a specific conformational epitope (Buell et al., 1998).

Recently, another mAb named BPM09 was shown as specifically targeting the E-200 epitope of nfP2X7, a nonpore functional form of P2X7 (Gilbert et al., 2017). Interestingly, this epitope is found only in cancerous cells (those from prostate, neuroblastoma, melanoma, breast, stomach, pancreas, colorectal, bladder, renal, etc.), not in normal cells, and this form is essential for cancer cell viability. This fact constitutes an advantage in terms of therapeutic perspective. Indeed, this receptor was shown to be upregulated in several cancers such as breast cancer, the third cause of mortality due to cancer in humans, and allows enhanced tumor growth (Gilbert et al., 2019). In the latter studies, a phase I clinical trial was conducted, yielding promising results using sheep polyclonal antibody against nfP2X7 (BIL010t)-based ointment as a therapeutic for basal cell carcinoma, which is an abnormal, uncontrolled growth or lesion arising in the skin’s basal cells; there are more than 4 million cases of basal cell carcinoma diagnosed in the U.S. each year (www.skincancer.org). Independent preclinical toxicology studies were performed in minipigs. These studies, conducted for 28-day and 90-day periods, demonstrated that the ointment was well tolerated (data not available). In addition, nanobodies against mouse P2X7 have been generated and characterized, which effectively blocked (13A7) or potentiated (14D5) gating of the channel in addition to nanobody Dano1, which specifically inhibited human P2X7 (Danquah et al., 2016). Indeed, nanobodies from six distinct families either blocked or enhanced both ATP-mediated and nicotinamide adenine dinucleotide (NAD+)-mediated activation of P2X7. The most potent blocker, nanobody 13A7 (IC_50_ = 12 nM), and the most potent enhancer, nanobody 14D5 (EC_50_ = 6 nM), were used in the study. However, all these studies were conducted using mouse models so far and further studies are needed to prove their efficiency in humans.

## Potassium Channels

The human Eag1 (hEag1), is a voltage-gated potassium channel named K_v_10.1 (Eag1) specifically expressed in the brain and in myoblasts (Gutman et al., 2005). This plasma membrane channel, encoded by the *kcnh1* gene, is modulated during the cell cycle (Brüggemann et al., 1997; Pardo et al., 1998). It is involved in several cellular processes such as cell excitability, memory processes, and cell proliferation (Saganich et al., 1999; Ufartes et al., 2013). In pathophysiology, it is overexpressed in more than 70% of tumors of several origin and so was suggested to be involved in tumorigenesis (Hemmerlein et al., 2006; Ouadid-Ahidouch et al., 2016). Thus, it constitutes a promising molecular target in cancer therapies or diagnostics.

In such a way, an anti-Kv10.1 mAb (mAb62) was used to target tumor cells *in vitro* using the human breast MDA-MB-435S cell line and *in vivo* on tumor models of nude mice (Napp et al., 2016). The authors demonstrated the specific binding and accumulation of the mAb62 in the tumors *in vivo* for at least 1 week due to the labeling with Cy5.5 fluorophore.

Another interesting mAb against hEag1 is mAb56. It recognizes two domains including E3 segment (residues 374 to 452) and tetramerizing coiled-coil domain (residues 872 to 932) (Ju and Wray, 2002; Schönherr et al., 2002). The latter allows specific binding to hEag1 and not to its closest homologue, hEag2 (73% amino acid sequence identity). The distinctive feature of this antibody is its antagonist effect on this channel, giving an IC_50_ value of mAb56 in HEK293 cells of 73 ± 47 nmol/L. Indeed, it selectively blocks hEag1 function. Inhibition of the potassium current mediated by hEag1 leads not only to a clear anti-proliferative activity *in vitro* but also to a decrease in tumor cell growth *in vivo* (ovarian, breast, cervical, colon, pancreas carcinoma, melanoma, and fibrosarcoma) (Gómez-Varela et al., 2007). Another antibody directed against the carboxyl (COOH)-terminal region of Eag1 (mAb33) had no effect on tumor growth compared to mAb56. Thus, the therapeutic potential of this mAb is very high.

## *N*-methyl-D-Aspartate Receptor

The *N*-methyl-D-aspartate receptor, also named NMDA receptor (NMDAR), is an ionotropic glutamate receptor found in nerve cells in which it acts as an ion channel protein that is nonselective to cations (Luini et al., 1981). It plays an essential role in the control of synaptic plasticity and memory function (Morris et al., 1990). Indeed Ca^2+^ flux through NMDARs is essential for long-term potentiation (LTP), which is a cellular mechanism for learning and memory (Furukawa et al., 2005; Li and Tsien, 2009). The modulation of such receptor affects persistent neuronal plasticity requiring *N*-methyl-D-aspartate (NMDA)-mediated transmission. It is well known that NMDAR antagonist impaired hippocampus-dependent learning (Morris et al., 2013).

Thomson and collaborators have developed an mAb, B6B21 (IC_50_ values of 1.6 µM), about 150 kDa, which targets NMDAR and acts as an enhancer of the receptor (Thompson et al., 1992). Actually, it enhances the opening of its ionic channel in a glycine-like fashion in displacing glycine. Because of this, it also improved LTP in hippocampal slices. Moreover, intraventricular infusions of this mAb in rabbit enhances acquisition in hippocampus-dependent tasks (Haring et al., 1991), such as hippocampus-related memory and cognitive function. B6B21 treatment resulted in a 45% reduction in the number of trials required to reach 80% conditioned responses and reductions in other operational measures of learning.

The development of peptide mimetics, derived from B6B21, may be potentially used as cognitive enhancers and protectors from hypoxic and ischemic insults exerted onto the neurons caused by stroke and epilepsy and also in cognitive disorder therapies. However, these peptide mimetics would likely exacerbate issues relating to hypoxia and ischemic insults. It should be taken into consideration that overstimulation of NMDAR known to impair early brain development *in vivo* observable in schizophrenia (Aida et al., 2012) is also involved in neuronal death hypoxia, ischemic injury, and stroke, due to inward calcium flux (Choi and Rothman, 1990). Hence, the use of such mAb could be have some deleterious side effects and have to be taken into account. On the other hand, Rapastinel (GLYX-13), a tetrapeptide derived from B6B21, displayed long-lasting antidepressant effects demonstrated in the human clinical trial (Moskal et al., 2017). Its clearance was rapid in normal human volunteers following i.v. administration *via* the antecubital vein over the dose range 0.5–25 mg/kg, and the plasma half-life was short, approximately 10 min. Thus, GLYX-13 appears to be promising because it seems to act rapidly with antidepressant effects that persist for approximately a week in humans and show no psychotomimetic side effects.

Another mAb that specially binds amino acids 657–678 of the extracellular domain of NR1 subunit has been developed (Amrutkar et al., 2012). Using resin-bound peptides, the authors identified a peptide sequence RNPSDK (amino acids 673–678 for the epitope) where the N-terminal arginine residue is essential for reactivity. It is situated in the extracellular S2 domain of the NR1 subunit. They showed that the antibody reactivity was dependent on a positively charged amino acid in addition to noncovalent backbone interactions (Amrutkar et al., 2012). However, neither further investigations nor characterization of this antibody was reported since then.

## Sodium Channels

In humans, the family of voltage-gated sodium (NaV) channels is composed of nine highly homologous NaV channels (NaV1.1–NaV1.9). They were shown to control the upstroke of the action potentials in excitable cells, whereas the NaV1.7 encoded by the *scn9a* gene seems to be involved in pain and itch modulation (Snyder et al., 2014). Indeed, it has been reported that a loss-of-function mutation in *scn9a* genes drive congenital inability to sense pain and anosmia, the inability to perceive odor or a lack of functioning olfaction (Cox et al., 2006; Weiss et al., 2011). On the contrary, gain-of-function mutations were shown to lead to episodic pain (Fertleman et al., 2006).

Moreover Lee and collaborators revealed that a mAb targeting the S3–S4 loop of domain II of NaV1.7, i.e., the voltage-sensor paddle, inhibits this channel with high selectivity (IC_50_ values of 30.7 nM). This mechanism lies in the stabilization of a closed state in a state-dependent manner (Lee et al., 2014a). That phenomenon was demonstrated by patch-clamp recordings using the whole-cell voltage clamp configuration on hNaV1.7 channels expressed in a transient manner in HEK293 cells. Using this mAb has allowed one to abolish inflammatory and neuropathic pain passing by central and peripheral mechanisms (intrathecal and systemic routes, respectively). Also, this antibody was shown to suppress nociceptive synaptic transmission in pinal cord dorsal horn as well as to reduce acute and chronic itch in mice (including chronic itch-potentiated synaptic transmission). All these effects are channel activity-dependent, as shown by the absence of effect of the control mAb that targets the S1–S2 loop of the channel. Thus, this mAb possesses considerable analgesic effects in mouse models of inflammatory and neuropathic pain including acute and chronic itch mouse models. This mAb could be employed in therapy for suppressing pain and itch (Lee et al., 2014a).

However, Liu et al. also showed that this antibody is not more efficient than the isotype-matched control antibody, IgG1 (Liu et al., 2016). In this study, they generated a recombinant SVmab1 (rSVmab1), using publicly available sequence information and the peptide VELFLADVEG, corresponding to a peptide loop between DII S3 and S4, as an antigen. This purified antibody did not specifically bind the S3–S4 loop and did not specifically inhibit the NaV1.7 current (Liu et al., 2016). Reductions in the Na V1.7 current were comparable when using an isotype control IgG1 or recombinant SVmab1 at 500 nM. These contradictory data could be due to the purification of the recombinant mAb from HEK293 6E cells that could induce differences in chain antibody sequences. Moreover, different glycosylation levels of the channel according to cell lines can lead to variable epitope accessibility.

## Concluding Remarks

Ion channels represent important therapeutic targets being mainly targeted by small molecules so far, which are always a compromise regarding their multispecificity and IC_50_’s. Antibodies are a growing alternative of important protein therapeutic drugs that can be employed as effective treatment alternatives for diseases and disorders such as cancer, inflammation, pain, impaired memory, and autoimmunity.

Antibodies, especially mAbs, constitute significant advantages because of their protein structure and biochemical properties that can be conventionally modified. Those are affinity, avidity, paratop 3D structure improvement, as well as Fc structure to engage complement reaction, all *via* antibody engineering and affinity maturation procedures. On the other hand, some disadvantages of using mAbs as therapeutics, such as their cost (high cost for production and low storage life), route of administration (only intraperitoneal or intravenous), and inability to cross the blood–brain barrier, can be impediments to antibody use.

Moreover, it has been demonstrated that mAbs can be used as experimental tools to study ion channel impact on the cell phenotype. Unfortunately, no antibody against ion channel to be used for therapeutic applications has been developed so far, which makes this approach continuously challenging. It can be explained that ion channels are actually quite difficult to target with Abs because their extracellular domains are very conservative among various species and thus are not antigenic. Another point other than peptide antigen is the case where conformational epitope is needed, which requires a significant quantity of the whole protein in its native conformation. Data summary on the already developed mAbs against ion channels and their prospective therapeutic applications is included in **Table 2**.

**Table 2:** Summarizing data on the developed mAbs against ion channels and their therapeutic potential.

Target	Channel type	Antigen	mAb function	Therapeutic potential	References
L- and N-type calcium channel	Voltage-gated calcium channel	Isoform 5 of α2δ1 subunits	Inhibitor used at 10 µg/ml (*in vitro*)	Hepatocellular carcinoma	(Zhao et al., 2013)
Orai1	Calcium release activated channel	Extracellular loop 2	Inhibitor (induces internalization) used at 10 mg/kg in mice three times per week in i.p.	Autoimmune disease	(Cox et al., 2013; Lin et al., 2013)
TRPA1	Transient receptor potential channel	Epitope unknown	IC50 value: 90 nM	Inflammatory and neuropathic pain	(Lee et al., 2014b)
TRPV1	Transient receptor potential channel	Prepore region	Antagonist used at 88.5–266.8 µg/ml	Thermal hyperalgesia in diabetic pain	(Klionsky et al., 2006)
Connexin 43	Nonselective	Second extracellular loop	Inhibitor used at 56–112 µg/ml (*in vitro*)	Detection of glioma cells in breast cancer	(Bao et al., 2011)
P2X3	Ligand-gated ion channel	Exact epitope unknown	Inhibitor used at 30 mg/kg in i.v.	Visceral pain	(Shcherbatko et al., 2016)
P2X4	Ligand-gated ion channel	Between 100 and 180 residue of the protein	Detection only	Neuropathic pain (allodynia)	(Igawa et al., 2013)
P2X7	Ligand-gated ion channel	Extracellular loop of the native protein	Antagonist 0.01–1.2 µg/ml for *in vitro* studies	Data nonavailable	(Buell et al., 1998)
Kv10.1	Voltage-gated potassium channel	E3 domain of the channel	Used at ≈833 µg/kg in mice in i.v.	Labeling cancer cells (showed in breast cancer)	(Napp et al., 2016)
Kv10.1	Voltage-gated potassium channel	E3 segment and tetramerizing coiled coil	Antagonist used at 25 mg/kg in mice in i.p. weekly	Pancreas and breast cancer	(Gómez-Varela et al., 2007; Ju and Wray, 2002; Schönherr et al., 2002)
NMDA receptor	Ligand-gated ion channel	Epitope not disclosed (dentate gyrus used as immunogen)	Enhancer	Cognitive disorder	(Haring et al., 1991; Thompson et al., 1992)
NMDA receptor	Ligand-gated ion channel	Amino acids 657–678 in the extracellular S2 domain of NR1 subunit	Recognition only	Autoimmune disease	(Amrutkar et al., 2012)
Nav1.7	Voltage-gated sodium channel	S3–S4 loop of domain II	Antagonist used at 10–50 mg/kg (i.v.), 50 µg (intraplantar) and 1–10 µg (intrathecal) in mice	Inflammatory and neuropathic pain	(Lee et al., 2014a)

NMDA, N-methyl-D-aspartate; TRPA1, transient receptor potential cation channel, subfamily A, member 1; TRPV1, transient receptor potential; vanilloid subfamily, member 1; i.p., intraperitoneal; and i.v. intravenous.

mAbs can also be used as conjugates, i.e., conjugated to toxic compounds or radioisotopes, or linked to the T-cell receptor [so-called chimeric antigen peceptor T-cell therapy (CAR-T) technology] to directly kill tumors or induce T-cell-mediated tumor cell destruction. They can be covalently linked to potent cytotoxic or cytostatic agents, including small-molecule drugs or inactive forms of a biological toxin. The antibody can deliver the toxin to the tumor cell that will endocytose the antibody–toxin complex and induce enzymatic cleavage and the release of the drug, yielding an active cytotoxic form and thus killing the tumor cell.

**Figure 2:**
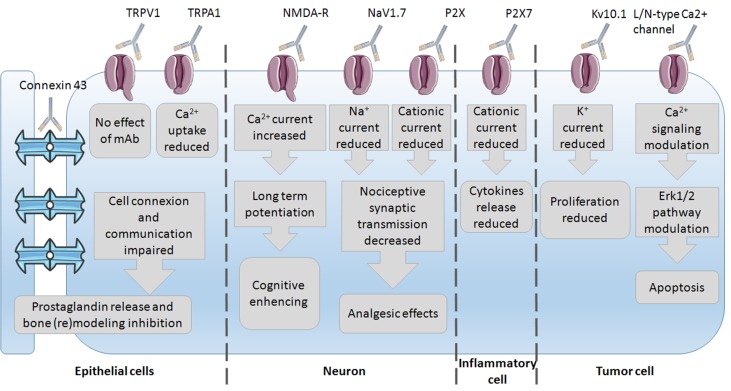
Insight into the mechanisms of mAbs’ action *via* their channels–targets onto the different cells.

Since mAbs have been used as experimental tools to study ion channel impact on the cell phenotype, several studies have already been conducted on the discovery of the underlying mechanisms of mAb action. The most common mechanisms of action observed are the reduction in ion current and thus modulation of the underlying pathways (**Figure 2**). These were mostly studied in epithelial, neuron, immune, and tumor cells with subsequent phenotype changes such as bone (re)modeling inhibition, cognitive enhancing, analgesic effects, reduction in proliferation, and induction of apoptosis. This wide variety of effects again highlights the importance of ion channels and their regulation of the phenotype control *via* mAbs. Little is known on mAb binding to any ion channel, and unfortunately, no crystal structure of antibody–channel complexes formed is available so far. For example, the mechanism of action of infliximab, a TNF-α inhibitor, primarily involves Fab-mediated antigen neutralization (Lee et al., 2017). The mechanism of infliximab action was shown to be the destruction of TNF-α producing cells through antibody-dependent cell-mediated cytotoxicity (ADCC). The Fc region of the infliximab immune complex binds to the Fcγ receptor (FcγR) on immune cells, leading to the lysis of target cells bound by the antibody Fab region, i.e., *via* ADCC.

Finally, 3D modeling of proteins for which X-ray crystallography is not available yet or computational approaches for protein function prediction using a combined strategy from multiple sequence alignment to molecular docking‐based virtual screening are of particular importance (Pierri et al., 2010). In the case of antibodies, molecular modeling is primarily employed for improving antibody–antigen affinity (or affinity maturation) using mutagenesis of antibody complementarity-determining regions (CDR) through *in silico* mutagenesis and the following assays on the bench (Hou et al., 2008; Pierri et al., 2016). Due to the available crystallization of three antibodies, the possibility of reconstructing a complete mAb sequence model based on the available templates 1IGT (Harris et al., 1997), 1IGY (Harris et al., 1998), and 1HZH (Saphire et al., 2001) is open to date for studying both antibody–channel interactions. Thus, the positive dynamics of anti-ion channel mAb research domains is likely to become exponential in the foreseeable future.

## Author Contributions

AH-G—writing of the basic version and preparing figures and tables. AH—doing all the revision amendments of the manuscript. NP—revising of the basic version and advices. VL—concept, supervision, and final version.

## Funding

This work was supported by Association de Recherche pour le Cancer, ARC (n°PJA 20161204599).

## Conflict of Interest Statement

The authors declare that the research was conducted in the absence of any commercial or financial relationships that could be construed as a potential conflict of interest.
